# HIV/AIDS and older adults in Cameroon: Emerging issues and implications for caregiving and policy-making

**DOI:** 10.1080/17290376.2018.1433059

**Published:** 2018-02-06

**Authors:** Perpetua Lum Tanyi, André Pelser, Joseph Okeibunor

**Affiliations:** ^a^ PhD, is a postdoctoral research fellow at the Department of Sociology, University of the Free State, Bloemfontein, South Africa; ^b^ MA (Sociology) and PhD (Sociology), is a Professor at the Department of Sociology, University of the Free State, Bloemfontein, South Africa; ^c^ Msc (Sociology) and PhD (Medical Sociology), is a Professor at the Department of Sociology, University of Nigeria Nsukka, Enugu, Nigeria

**Keywords:** HIV/AIDS, older adults, African family, caregiving, Cameroon

## Abstract

The burden of human immunodeficiency virus (HIV) infection and acquired immune deficiency syndrome (AIDS) on the elderly population in three divisions within the Northwest Region of Cameroon was examined. Data for this paper were extracted from a larger study which had been conducted concerning the burden of HIV infection and AIDS on the older adults in the Northwest Region of Cameroon. Using in-depth interviews (IDIs) and focus group discussions (FGDs), data were collected from 36 participants who were purposively selected from the three divisions which had been chosen randomly. 6 FGD sessions were held with 30 women aged 60 years and above and who were affected by HIV infection and AIDS, while IDIs sessions were held with 6 male community leaders. The results revealed that HIV infection and AIDS has added another dimension to the role of older persons. HIV infection and AIDS affects older people in diverse ways, as they have to look after themselves, their sick children and are often also left to look after their grandchildren orphaned by HIV infection and AIDS. These emerging issues in their lives make them vulnerable to health, social, economic and psychological challenges, and place a burden on them as caregivers instead of being cared for in their old age. Apart from increased direct expenditures, taking care of victims of HIV infection and AIDS requires older people to stay away from social, religious and community activities. The results showed that the loss of a child to HIV infection and AIDS affects the economic/financial well-being, participation in social/religious interactions as well as the community activities of older people participants. The implications of these findings for caregiving and social policy are discussed.

## Introduction

A major challenge facing the African family structure today is the increasing burden of human immunodeficiency virus and acquired immune deficiency syndrome (HIV/AIDS) on the elderly in African households (Casale, 2011). Sub-Saharan Africa is home to only about 13% of the global population, but it harbours nearly 70% of people living with HIV (PLWHA) (Population Reference Bureau, 2015; UNAIDS, 2016). Literature (Lekalakala-Mokgele, 2011) reveals that the epidemic has had a devastating impact on older adults. In Sub-Saharan Africa, for instance, about two million AIDS-related deaths are recorded annually and at least 13 million children have lost either one or both parents as a result of the pandemic (African Union, 2012; Munthree & Maharaj, 2010; Tanga, 2013; UNAIDS/WHO, 2010). The impact of HIV/AIDS, as well as the rapid growth of population ageing in Africa, has interlocked to not only create new emerging issues but also added new dimensions to the roles that older persons play in society. One of the major ways in which the HIV/AIDS epidemic has affected the lives of older persons is through caregiving (Gadling-Cole, Crewe, & Joyner, 2011; Schatz & Seeley, 2015; Zimmer & Dayton, 2005). Older adults have to care for themselves as well as their sick children and are often also left to look after their grandchildren orphaned by HIV/AIDS. These realities make them vulnerable to health, social, economic and psychological challenges, and place a burden on them as caregivers instead of being cared for (Mugisha et al., 2013; Mugisha, Schatz, Seeley, & Kowal, 2015; Scholten et al., 2011; Tanga, 2015).

HIV/AIDS in Cameroon is generating orphans quicker than family efforts can cope with (Mbanya, Sama, & Tchounwou, 2008). This is because HIV/AIDS mostly attacks the reproductive and economically active section of the population, changing family composition by creating elderly headed families (AVERT, 2009; UNAIDS, 2010). Demographically, this has resulted in a greater number of elderly people who have now taken over the responsibility of caring for children orphaned by HIV/AIDS. This has direct effects which have manifested in a set of interrelated, economic/financial, and social/religious dimensions and community challenges for elderly parents (Awuba & Macassa, 2007; Tanyi & Keet, 2016). Consequently, the roles of the elderly are seen to be changing to that of caregivers instead of those being cared for (Bock & Johnson, 2008; Help Age International, 2007; Ogunmefun & Schatz, 2009). Despite these changes, their role as caregivers is most often not properly examined, analysed and documented (Lee, Li, Jiraphongsa, & Rotheram-Borus, 2010; Ssengonzi, 2009; Williams, Knodel, Kim, Puch, & Saengtienchai, 2008).

Studies carried out by Knodel, Watkins, and Van Landingham (2003) and Knodel and Kespichayawattana on HIV/AIDS and older adults from an international perspective reveal that the impact of the worldwide HIV/AIDS pandemic on persons aged 60 and over has received relatively little consideration. A study of the complex association between economic outcomes and caregiving for adult children who are living with or have died of AIDS-related conditions by Knodel, Kespichayawattana, Saengtienchai, and Wiwatwanich (2010) and Chepngeno-Langat, Falkingham, Madise, and Evandrou revealed that older adults had negative economic outcomes due to taking care of their ill children. Another study by Ardington et al. (2010) and Hecht et al. (2010) addressed both economic and health impacts of caregiving to orphans and results revealed that there were significant transfers of funds from the government and families to older adults caring for orphans, but the older adults still had health and financial problems as a result of taking care of their ill children. Studies on community reaction related to older aged caregivers and their families (Knodel, Williams, Kim, Puch, & Saengtienchai, 2010; Tanga & Tangwe, 2014; Tanyi & Okoye, 2014) revealed a mixture of positive and negative reactions present but that positive support from others in the community was often dominant. This finding is contrary to common portrayals that predominantly emphasis the negative aspects associated with HIV/AIDS (Li et al., 2008; Møller & Erstad, 2007).

Although extensive research has been carried out in Cameroon on various issues relating to HIV/AIDS (Boyer et al., 2010; Mbanya et al., 2008; Musoko, Macauley, Zoungkanyi, Bella, & Koulla-Shiro, 2007), very little research has, however, been done on the impact of HIV/AIDS on the economic/financial, social/religious and community life of older adults and their children who are affected with HIV/AIDS. The limited focus of research to date in Cameroon detracts from a fuller understanding of the social context of the epidemic in the country. In order to fill this gap, the major objective of this study was to examine the burden of HIV/AIDS on the older adult population in the Northwest Region of Cameroon. For purposes of this study, an ‘older adult’ is regarded as a person who is 60 years and above and who had lost an adult child to HIV/AIDS, taking care of children orphaned by HIV/AIDS or whose child was currently HIV positive and receiving availability of antiretroviral therapy (ART). As a result, this article reports on the social, psychological and economics effects of HIV/AIDS on the older population of the Northwest Region of Cameroon and identifies appropriate intervention strategies to mitigate the impact of HIV/AIDS on the elderly in Cameroon.

Theoretically, this study contributes to the growing field of social gerontology and HIV/AIDS by providing data on the burden of HIV/AIDS on the older adult's population of the Northwest Region of Cameroon. Since very little research has been conducted on this topic, this paper contributes to filling a gap in the existing body of literature on elderly people and HIV/AIDS, particularly in Cameroon. This study will also serve as a reference point to policy-makers who want draw up policies on issues concerning the burden of HIV/AIDS on the elderly.

## Methods

### Study design

The data for this paper were extracted from a broader study which was carried out in three divisions in the Northwest Region of Cameroon and which aimed at studying the burden of HIV/AIDS on the elderly population. The broader study employed both quantitative and qualitative methodologies, which examined the economic effects associated with the death of an adult child with HIV/AIDS, the well-being of grandparents three years ago versus today (when the data was collected), the grandparents’ participation in religious/social activities as well as their participation in community activities.

For purposes of this paper, we adopted a qualitative research design, aimed at understanding the burden of HIV/AIDS on grandparents who are 60 years and older. The rationale for choosing a qualitative approach was provided by the aim of obtaining a comprehensive description and elucidation of the actual experiences of elderly parents who are affected by HIV/AIDS.

Ethical approval for the study was received from the institutional review committee board at the University of Nigeria Nsukka. The reason being that the proposal for this study was conceived in Nigeria, so it had to pass through the institutional review committee board of the University of Nigeria Nsukka. Approval for the study was granted on 23 February 2013, while informed oral consent was obtained from community leaders in Cameroon to conduct the focus group discussions (FGDs) and in-depth interviews (IDIs). This is because the community leaders will not allow any research in the community unless they (the leaders) have given permission for such research to be undertaken. The researcher was asked the purpose of the research and it was explained to the community leaders. Participants were assured of confidentiality, anonymity and the right of refusal to participate in the study without any penalties inflicted upon them. Also, oral permission was sought from the participants to audiotape the sessions.

### Conceptual framework

Conceptual frameworks for analysing potential pathways through which the illness and death of a HIV-infected adult child could impact older-age parents in low and middle-income countries were developed prior to widespread availability of ART (Knodel et al., 2003; Van Landingham, Im-em, & Saengtienchai, 2005). This study adopted the conceptual framework titled ‘potential pathways to adverse impacts of the HIV/AIDS epidemic on parents of infected adults prior to widespread availability of antiretroviral therapy’ developed by Knodel et al. These frameworks guided the formulation of the research questions addressed in this present study of how the death of an adult child to HIV/AIDS has affected the lives of the older adults.

**Fig. 1.**
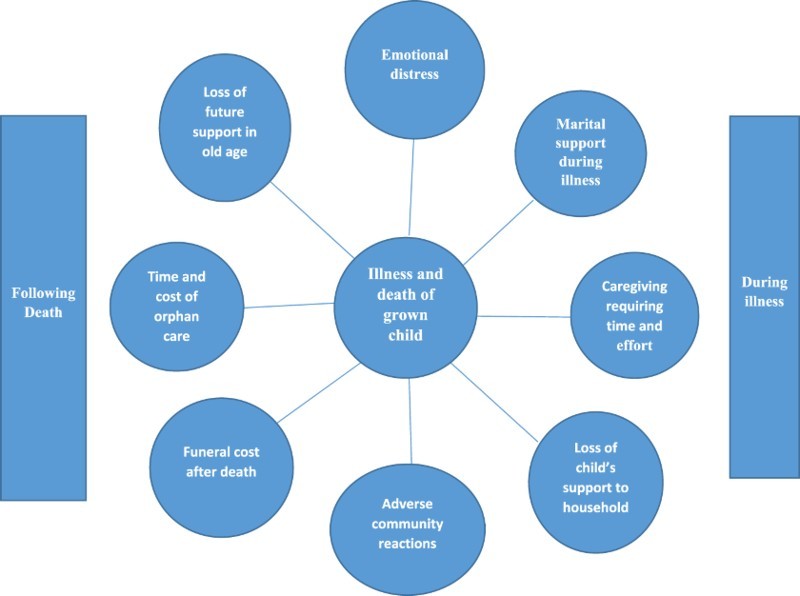
Potential pathways to adverse impacts of the HIV/AIDS epidemic on parents of infected adults prior to widespread availability of ART (Knodel et al., 2003; Van Landingham et al., 2005).

Fig. 1 provides a general frame of reference for considering the main potential pathways through which the HIV/AIDS epidemic has affected older adults. How much these potential impacts actually occur remains largely for systematic empirical research to determine. They are likely to be context sensitive and thus to vary across different countries. Of the all the pathways considered, in Fig. 1 (caregiving, providing financial or material support during the time the adult child is ill, sponsoring the funeral of the deceased child, and fostering grandchildren) are applicable to this study.

#### Caregiving

Proving care to an ill adult child can affect the well-being of the older-age parent. As caregivers, older parents can suffer considerable psychological pain witnessing the suffering and death of their child. Caregiving demands enormous time and effort, especially at the terminal stage of AIDS. Older adults may feel upset over negative reactions from members of the community who associate the caregiving role with contamination by HIV. Adverse financial consequences could result when caregiving competes with time needed to earn a livelihood. Some caregiving tasks, such as lifting the ill adult child, may lead to physical strains. Also potential exposure to the opportunistic diseases (especially TB) exists if not to HIV (for which the risk of infection through caregiving is extremely low). Caregiving also can divert time from social activities or lead social shunning by others who have misgivings about being near the caregiver or the adult child with AIDS. Intra-familial relations become strained when caregivers perceive inequities in the contribution of other family members.

#### Providing material support

Older parents may help with medical and living expenses associated with the illness or with the upkeep of the ill child's dependents. To do this, parents may go into debt, deplete their savings or sell assets to cover these extra expenses. Such contributions can lead to anxiety among parents over their own economic security. A parent may need to take on extra work to cover the costs, which, if physically taxing, could affect the health of the parent.

#### Funeral expenses

Funerals often involve significant costs for surviving parents and affect their economic well-being in the same ways as the costs involved prior to the death. If community members avoid attending the funeral or act in offensive ways at the funeral (e.g. refuse food or avoid being near the corpse), parents suffer socially and emotionally.

#### Taking care of grandchildren

Older adults may inherit responsibility for AIDS orphans with obvious financial implications. Emotional strains may result from negative community reaction towards the fostered grandchildren or worries about the costs of childcare. Physical strain and exhaustion can result from additional work required to cover these costs. Foster care may diminish social activities, lead community members to avoid the family for fear that the grandchild is infected, or strain intra-familial relations if conflicts over custody arise or foster parents judge other family members as negligent in shouldering adequate responsibility (Knodel et al., 2003; Van Landingham et al., 2005).

#### Death of adult child to HIV/AIDS

An adult child's death to HIV/AIDS can devastate parents and lead to lasting grief (De Vries, Lana, & Falck, 1994). Anxiety can ensue when parents depend economically on the child or had planned to do so. Current economic well-being may decline if the child who died contributed financially to parental support or assisted in household economic activities. In addition, parents lose any potential support that the deceased child might have provided in their old age.

#### Community reaction

Negative reactions of others in the community towards the parents, either during the time of the child's illness or following the death, could cause psychological, economic and social distress. For example, a shopkeeper whose child dies of AIDS may lose business because customers fear contagion. If other family members have a similar fear, strained intra-familial relations may result.

### Study setting

The study was conducted in the Mezam, Menchum and Donga/Mantung Divisions in the Northwest Region of Cameroon. The Northwest Region is made up of seven Divisions, of which Mezam, Donga/Mantung and Menchum are inclusive. A 2010 study by UNAIDS and Demographic Health Survey found that the Northwest, East and Southwest Regions have HIVAIDS prevalence rates of 8.7%, 8.6% and 8.0%, respectively. In great contrast, the North and Extreme North Regions have infection rates as low as 1.7% and 2.0%, respectively. Since WHO/UNAIDS (2010) identified the Northwest Region as one of the regions in Cameroon with the highest HIV prevalence rates, it was decided to select this region for the study.

### Selection of participants

This selection of the Northwest Region was based on the reported rate of HIV, which showed a high prevalence in the three divisions. One community was purposively selected from each of these divisions. With the help of local contact persons who were health workers, community leaders who were not part of the local contacts and research assistants, 36 eligible older adults were selected for the qualitative component of the study. The health workers from their records, helped to identify the older adults who had lost a child to HIV/AIDS and who were taking care of children suffering from HIV/AIDS. The research assistants helped in the collection of data. Community leaders gave us their opinions about issues surrounding older adults affected by HIV/AIDS.

The 36 participants were made up of 30 elderly women who participated in the FGDs and 6 men who were community leaders and who participated in the IDIs. The rational for selecting only women for the FGDs resulted from the fact that most men refused to participate in the FGDs. They listed reasons such as not having time and that they did not want to be reminded of the death or sickness of their adult children. The decision was then taken to eliminate male participants from the FGDs and focus on women only.

Six FGDs sessions (two per Division) were conducted in the three selected Divisions. Each focus group consisted of five participants. The older adults who participated in the FDGs were selected from a pool of potential participants who met the following criteria: (i) living in any of the three divisions, (ii) being an adult who is 60 years and above and who had lost an adult child to HIV/AIDS, (iii) taking care of children orphaned by HIV/AIDS or (iv) whose child was currently HIV positive and receiving ART. Six male community leaders, two from each of the three divisions were purposively selected from the communities to participate in the IDIs. Six interview sessions were held in the homes of the community leaders. The selection of males was motivated by the fact that there were no female community leaders in the three selected divisions.

### Generation of data

The researchers developed an FGD guide and a semi-structured IDI schedule to gain insights into the shared experiences of the participants, in order to understand the changes in their lifestyle as a result of the effect of HIV/AIDS.

IDIs and FGDs constitute an important means by which information pertaining to opinions, personal experience and perceptions may be extracted. According to Creswell (2014), other methods of collecting data are unlikely to yield the same depth of information. The questions that were asked were based on the participants’ perceptions of their burden because of HIV/AIDS, their economic/financial well-being, participation in religious, social and community activities. The participants in the IDIs responded to these questions during one-on-one interviews with male research assistants in Pidgin English. All the interviews took place in the homes of the community leaders as requested by them, and each was approximately one hour in duration.

The first author, with the help of research assistants, conducted the FGDs. These lasted an average of one hour and were typically conducted in the homes of one of the participants, who was the most elderly and who volunteered for her home to be used. An honorarium to the amount of 3400 CFA Franc (approximately US$7) was given to each of the focus group participants as a token of appreciation for their time and willingness to participate in the study.

### Analysis of data

All the FGDs and IDIs were transcribed and translated verbatim from Pidgin into English with the help of the same research assistants who had conducted the interviews and the FDGs, and who also served as note takers. In order to achieve ‘immersion in the data’, the transcribed texts were read by a colleague who understood the variant of Pidgin English spoken in the communities where the discussions took place. This enabled the authors to ensure that no information was lost in the translation. A final version of the transcript was obtained based on the inputs of the research assistants and the colleague who read the transcribed notes.

The next step was to describe the significant components and recurrent themes which emerged. The descriptions from which the various themes were identified were synthesized in order to be able to substantiate statements by means of direct quotations from the responses given by the participants during the interviews. The themes included the following: the Economic/Financial well-being of older adults; participation in religious/social activities and participation in community activities by parents taking care of PLWHA. In order to accurately portray the sentiments of the participants, verbatim quotes were used in some instances.

### Trustworthiness

The trustworthiness of the findings in this study was established as a result of the integrity of the data, which was achieved by obtaining a balance between reflecting objectively on the part of the researchers and the subjective responses of the participants in order to provide a clear communication of the findings in the manner recommended by several authors (Arczynski & Morrow, 2016; Arczynski, Morrow, & Englar-Carlson, 2016; Campbell, Olson, Keenan, & Morrow, 2017; Hoover & Morrow, 2016; Levitt, Motulsky, Wertz, Ponterotto, & Morrow, 2016). The dependability of the data determines the integrity of the data. The rational for selecting only women for the FGDs resulted from the fact that most men refused to participate in the FGDs. They listed reasons such as not having time and that they did not want to be reminded of the death or sickness of their adult children. The decision was then taken to eliminate male participants from the FGDs and focused on women only.

In order to ensure accurate interpretations with data, we included some of the direct quotations from the verbatim transcriptions of the audio recordings of the interviews with the participants. We also engaged in active and sustained reflection while reading and analysing the data. This enabled us to strike a balance between the responses of the participants, on one hand, and the meanings which we interpreted from them, on the other. In addition, to ensure that objective reflection and subjectivity were balanced, our individual interpretations and those of the research team were used when they seemed most appropriate. Throughout the writing process, we constantly referred to the research questions in order to ensure that our claims were adequately supported by evidence.

## Findings

In this section, the results of the qualitative analyses are presented under different subheadings, namely key socio-demographic characteristics of the participants, economic/financial well-being of the participants, participation in religious/social activities and participation in community activities of the participants.

### Socio-demographic characteristics of the participants

The characteristics of the female participants in the FGDs show that 60% were between 60 and 69 years, 63% of them earned less than $100 dollars per month, about 50% of them did not have any other living children, about 40% had lost a child to HIV/AIDS and 80% had at least one child who is currently suffering from HIV/AIDS. Eighty per cent of them were leaving and taking care of grandchildren orphaned by HIV/AIDS. The characteristics of the male participants in the IDIs show that three were 60 years, two were 71 years and one was 75 years of age, four of the six earned 100 dollars and above per month, five of them had other living children, none of them had lost a child to HIV/AIDS and none had a child suffering from HIV/AIDS.

### Economic/financial well-being of parents

#### Taking care of grandchildren

To enable us to investigate the economic/financial effect associated with a child's death/illness from HIV/AIDS, participants in the FGD sessions were asked about their financial situations, whether their financial situation had changed since they lost their child to HIV/AIDS or since their child took ill of HIV/AIDS and the reasons for the changes. The participants made it clear that there were definite changes in their lives as a result of the death of a child due to HIV/AIDS and the illness of their child to HIV/AIDS. The burden of caring for grandchildren orphaned by HIV/AIDS also has an impact on their financial situation. When asked about their financial situation before and after the death of their child to HIV/AIDS, these were some of the responses they offered:

*Well, so many years ago when things were still good we really lived a good life, my child would take me to church, she would take me to visit my friends. But everything has changed since she became sick. We hardly go to church and we don't have so much money to spend again. We even have to borrow money at times to buy her drugs and the type of food she eats. Things are not the same again. We don't have money*. (A 65-year-old mother of a child on ART, an urban dweller in Mezam division)

*I am caring for three girls and three boys. They are sleeping together in one room. I need separate bedrooms and beds for the girls and boys. I wish to put a TV in the room. I don't have enough money to buy them*. (A 70-year-old mother of a deceased daughter to HIV/AIDS, an urban dweller in Menchum division)

Caring responsibilities also exert financial costs to elderly people who are taking care of either their sick child or grandchildren left behind by their deceased parents. Another participant captured it this way:

*I have sold all my property to take care of my sick child and now he is dead, I don't have any money again to take care of these children he left with me. The wife is also sick and I don't think she will last up until end of the month*. (A 65-year-old mother of a deceased son, an urban dweller in Donga/Mantung division)

The data showed that the older adults often get distressed about not being able to provide enough food, and clothes, or being able to meet children's educational and health needs, except for a few cases in Mezam division where the elderly were able to properly take care of their grandchildren. This is because some of the older adults are still working and others who are retired had a pension. The financial burden on older adults who care for grandchildren orphaned by HIV/AIDS is indeed a daily reality for the participants and a challenge that defines their economic existence.

Taking care of a grandchild orphaned by HIV/AIDS can only occur if the deceased parent had surviving dependent children. Results from the FGDs reveal that more than half of the adult children who died of AIDS had surviving children. This high proportion could be attributed to the fact that a majority of the deceased parents were married and had children. Moreover, most of the deceased parents who died had more than one child. In the Northwest Region of Cameroon, it is quite uncommon to find two grandparents who are still alive, which makes the burden of caring for grandchildren even more difficult for the surviving grandparent who often is a woman.

Participants in the FGDs were asked how many children they had. One of the participants captured it this way:

*I had five children and now only one is still alive. All the other ones are dead. They died of that thing. (Most of the time elders refer to HIV/AIDS as ‘that thing’). This child who is alive is always very sick and the doctor says he has HIV/AIDS. We spend so much money on buying medications for him. I have five grandchildren in the house with me. We all do sell fruits to pay their school fees*. (66–year-old grandmother of deceased children to HIV/AIDS and a child on ART, a rural dweller in Menchum division)

In the FGDs, it also turned out that the participants often had to look after their grandchildren following the death of their own children:

* … I have six grandchildren staying with me, three are from my son and the other three are my sisters’ children. The children know that I do not have anything, so we all go to the farm. When they go to school, I go to the farm with the young one, and when they come back from school, they join us. They are doing very well in school. We all work to pay school fees for everybody. They also do jobs for people who need them to work on their farm. They make me very happy. The young boy bought the television we are using in the house. I am happy with my grandchildren*. (67-year-old grandmother of deceased children to HIV/AIDs, a rural dweller in Menchum division)

Another participant also remarked that:

*I have two grandchildren. Their father and mother died when they were very young and I have been taking care of them. The first one graduated from university last year and the other one will graduate this year. It was not easy for the kids and me, but I am happy the Lord is helping us. I was a teacher, but I am retired now. I paid all their fees and bought everything for them when they were growing up. They are a source of happiness for me. I miss my child at times, but when I look at the two children, I praise God*. (70-year-old grandmother, of deceased children to HIV/AIDS, an urban dweller in Mezam division)

Participants were further asked where the grandchildren were living. Some of the responses included the following:

*I lost my son last year and just two months ago my daughter was brought back from the city dead. I am staying with all my grandchildren they left behind. The grandchildren are six in number. Four from my son and two from my daughter. I am not working. It is so difficult for me. I feed them and buy clothes for them. I try to pay their school fees any time I have money*. (60-year-old mother, of deceased child to HIV/AIDS, a rural dweller in Mezam division)

Another also remarked that:

*I live with my daughter's children in one room. There are three of them in number. I am a widow and do not have anything to do. I only go to the farm with my grandchildren and that's how we get money to eat and pay school fees. We all work on the farm and go to the market to sell goods. I only advise them to be of good manners and they are very good children. They make me happy all the time. I am praying that God should help them finish school and get good jobs*. (65-year-old mother of deceased daughter, a rural dweller in Donga/Mantung division)

#### Changes in the quality of life of grandparents

In the FGDs sessions, we asked participants why they were living with their grandchildren and whether they were taking care of their grandchildren. We also asked them about their financial situation before they started taking care of their grandchildren, whether they owed any money to anyone in the community, whether they had financial problems as a result of them taking care of their grandchildren and whether they had received any form of support from community members during the illness or death of their children (Appendix 1). Some of them responded as follows:

* …  I have four grandchildren staying with me, their mother died two years ago. The husband abandoned her when she fell sick. So now I have to look at my grandchildren because they don't have any other person except me*. (64-year-old mother of deceased child, urban dweller Mezam division)

* …  When my son was alive, he gave me money to take care of my needs, he took me to the hospital for a check-up. I was very comfortable, I had no problem at all, I was happy, but now that he is dead I have so many financial problems. At times when I am sick, I don't have money to go to the hospital. His wife is very sick now. I have these three grandchildren and the wife of my son to look after. I am not happy at all, I don't have anyone to help me*. (She started crying). (68 years old mother of a deceased son, an urban dweller in Menchum division)

*The community members came to visit us, and most of them gave me money. Some of them gave me food and clothing for my sick child. Many of them brought firewood for us*. (68 years old Mother of a deceased son, an urban dweller in Donga/Mantung division)

#### Increased economic dependency on community members

It is clear from the responses above that the death of their children as a result of AIDS has left these elderly women with serious financial difficulties to cope with and has caused a drastic decline in their quality of life. This, in turn, has increased their economic dependency on community members. To get the views of the community leaders concerning care for people affected by HIV/AIDS, we asked them whether there were any community efforts to care for people affected by HIV/AIDS. Two community leaders responded as follows:

* …  As a community, we try our best to send food, or visit when we fine someone who is sick of HIV/AIDS. We attend funeral and help to bury the deceased person if the family cannot do it. It is our culture to stay with people when they are bereaved*. (69-year-old community leader in Donga /Mantung division)

*In this community, we try to help ourselves. We stay as brothers and sisters and if there is any problem, we come together and help one another. We are begging the government to do something about this sickness. They should try to get the cure for it*. (67-year-old community leader in Mezam division)

We also tried to find out whether there were any community efforts to care for the children orphaned by HIV/AIDS. The following responses serve to illustrate the vulnerability of the orphaned children:

* …  We do not have any money to pay school fees for the children left behind by the dead children in this community*. (78-year-old community leader, a rural dweller in Menchum Division)

*We have tried to help some children whose parents are dead. Now what we do is that when their parents die we take them to the Reverend Fathers and Sisters. Some of them are sent to school but some go to learn any trade of their choice. At times, the Reverend Father refuses to take some of them. There is one woman who comes from the city to give them food and clothing*. (67-year-old community leader, a rural dweller in Mezam division)

Other community leaders confirmed the responses above. For instance, a 68-year-old community leader in Mezam division mentioned that they did as much they could to help the elderly people affected by HIV/AIDS. They tried to tell community members not to avoid those affected or infected, but that it was a difficult situation because people were afraid of contracting the virus if they went close to the sick man. Another had this to say:

* …  In our tradition, when someone dies in a community we go and stay with the member to consul him or her, irrespective of the type of sickness the member's person died of*. (70-year-old community leader in Menchum division)

#### Increase in personal debt

Participants were also asked whether they were indebted. In all three divisions, we found that the participants had serious debt issues as a result of borrowing money to cover the expenses of treatment, caretaking and funerals. The rate of indebtedness was higher in Mezam division than the other divisions. One participant explains:

*My son and my daughter died almost the same year. I have eight grandchildren, I am staying with. My daughter's husband is dead, my son's wife is sick too, but her brothers came and took her away. I am owing so many people in this community. I am even owing the hospital. When my child was sick I took her there and I did not have money to pay the bills, so they collected my identity card. I cannot even move around now because I don't have the identity card. Police will arrest me. I cannot go to anyone now to help me in this community because they will not lend me money*. (68-year-old mother of a deceased son, an urban dweller in Mezam division)

Yet another mother whose experience of taking care and losing a child to HIV/AIDS had the following experience to share:

*My daughter was in the hospital for more than three months before she died. I did not have money to pay for the entire hospital bill. The hospital authorities refused to release her body. I came back home to look for money, but no one gave me money because they said she was a bad girl and died of a bad sickness. The body is still in the hospital. I do not have anyone to help me pay the bills so that I can take my child's body and bury her*. (60-year-old woman, a rural dweller in Donga/Mantung division)

Funerals are very important social events in Cameroon and reflect the prestige and reputation of a family within the community. Funerals usually last at least several days and involve treating guests with refreshments and meals. The expenses for funerals are incurred all at once, unlike the cost of care and treatment that may be spread out over the full period of illness.

Some participants had very bad experiences with regard to funeral expenditures, such as the one shared by a 61-year-old woman:

*My girl was staying in the city with my sister when she became sick and they sent her to the garage to stay and sent for me. When I arrived, I asked for my girl and my sister's son took me to the garage and showed me my girl. She was just sleeping there helpless. I asked her what the problem was. She could not talk so I went to my sister's office and asked her and she told me that she had AIDS and she could not allow her to stay with her children in the house. I begged her to give me some money to take my child to the hospital and she gave me 200 FRS CFA. That was not enough to pay a taxi fare, so I paid the wheel- barrow man who carried her to the hospital. I left her and went back to the village to look for money, but nobody was willing to give me money. I spent two days in the village. By the time I returned she was so bad then she died. She was buried in the hospital cemetery because I did not have money to bring her corpse back. There is still no funeral for her yet, because I don't have money.*

Participants also reported that during funerals, the female relatives of the deceased are required to come and stay with the family of the deceased for some days before going back to their homes. However, this practice was reported by some participants to be a further financial burden because they had to feed these relatives and even provide transportation for some of them.

### Psychological well-being of older adult parents

Taking care to an HIV/AIDS adult child can affect all aspects of the well-being of the older adult parent. As caregivers, older adults could suffer considerable psychological pain witnessing the suffering and dying of their adult child to HIV/AIDS. Caregiving requires enormous time and effort, especially at the terminal stage of AIDS. Older adult parents may feel upset over anticipated or actual negative reactions from members of the community who associate the caregiving role with contamination by HIV. Adverse financial consequences could result when caregiving competes with time needed to earn a livelihood. Some caregiving tasks, such as lifting the ill adult child, may lead to physical strains. Also potential exposure to the opportunistic diseases (especially TB) exists if not to HIV (for which the risk of infection through caregiving is extremely low). Taking care of an adult child suffering from HIV/AIDS and also grandchildren also can divert time from social activities or lead social shunning by others who have misgivings about being near the caregiver or the adult child with AIDS. All the respondents in the three divisions responded that they had so much pain seeing their child suffer and die of HIV/AIDS. The death of their adult child to HIV/AIDS has affected their entire life style.

### Participation of participants in religious/social activities

An adult child's illness and death from HIV/AIDS can adversely affect the social and religious well-being of the elderly. Most elderly parents spend considerable time either taking care of their sick children or taking care of the grandchildren orphaned by their deceased children. This may affect the social/religious activities of the elderly in the community.

Participants in the FGDs were asked whether they participated in religious and social activities (such as going to visit friends and attending social functions like weddings, parties, child dedications). If so, they were asked to explain the type of social/religious activities they participate in. They were also asked about the frequency of their attendance of such activities.

Most often community members avoided social contact with people with HIV/AIDS and their families, probably due to fears of infection. Only in a few cases, though did participants explicitly stated that community members clearly stopped visiting them. In many cases, neighbours avoided direct social contact only with the PLWHA. However, a few participants mentioned that neighbours were also afraid of being infected by other members who had close contact with the PLWHA. They, therefore, avoided contact with the parents, siblings or children of the PLWHA. Participants also reported that, in some cases, they deliberately kept their distance for fear of rejection, even when they had experienced no obvious signs of rejection by the community. One participant captured it this way:

*When my boy came back from university, he was very thin and he told me he was sick. He told me that the doctor told him that he was HIV positive. I did not believe him so we went to the general hospital and it was confirmed. When he started treatment, many of my friends stopped coming to my house. Most of them stopped talking to my son. At times when we go to church, everybody will leave the bench for just two of us. He is my only son, I cannot abandon him. I take him to the hospital every time he wants to buy his drugs. I do not feel good about it, but the Lord is my strength*. (67–year-old mother, an urban dweller from Mezam division)

Many of the participants reported that they have reduced their level of social interaction, because of either caring for grandchildren left behind by a deceased parent or because of caring for a sick child. Said one woman:

* …  Before my child took ill, I was a member of Saint Jude. I used to go to church for prayers and do some work in the church, but since he took ill I do not go again. At times, my group members come to pray for us in the house*. (70-year-old mother, an urban dweller in Menchum division)

Another old woman had this to say:

*My children will not allow me to go elsewhere; the grandchildren are all over the place. Even though there is someone to help but I still do not have the heart to leave them and go elsewhere. I am the only person they have now. I only send my contribution through the house girl. I stay at home to take care of my sick child and my grandchildren*. (66-year-old mother, an urban dweller in Mezam division)

The illness and death of an adult child with HIV/AIDS might cause parents to stop attending meetings or joining social groups. While some participants said that they had no time to attend social/religious activities anymore because they were either taking care of their sick children or children orphaned by deceased children, some still had time to attend such activities. In only a few cases did participants say they attended meetings to keep themselves happy and to distract themselves from their existing problems.

### Participation of participants in community activities

Participants in the FGD sessions made us understand that it was compulsory to participate in community activities. The main reason was that if they did not participate in community activities, the community members would not support them in times of trouble. For that reason, participants were asked whether they participated in community activities and to mention the types of activities they participated in.

A 65-year-old woman in Mezam division reported:

*I attend community activities. I am not strong but I must attend, so that if I have problems the community members will assist me. If I don't attend they will not assist me in the time of trouble.*

Another woman said:

*I help to sweep the market square when there is community labour. I don't go all the time because of my son's health, but I try to attend so that when I have a need, the community members will help me*. (65-year-old mother of children on ART, an urban dweller in Donga /Mantung division)

In the interviews with community leaders, we tried to find out their views about participation in community activities by older parents who had lost an adult child to HIV/AIDS. This is because in our discussions with the older parents they made us understand that participation in community activities was compulsory. We also tried to determine if there was any form of sanction or penalty for people who discriminated against those who had children suffering or had died of HIV/AIDS (Appendix 2).

A 67 years old community leader in Mezam division reported:

*Every person in my community must participate in community activity. That is the time we come together to clean the community. Anyone who does not come out will be punished. We will not help the person when the person has any trouble. We will not visit the person when the person is ill.*

Another community leader said:

*The reason why we ask people to come out for community activities is because, some of the people in the community who have lost children to HIV/AIDS feel very shy and so they stay away from people. We make the participation in community activity compulsory so that they will not isolate themselves. It is also a way of making them feel free with other members of the community*. (70-year-old community leader in Menchum division)

*A 70 years old community leader in Mezam division made us to understand that they were very strict rules concerning anyone who tried to discriminate against older parents who had lost an adult child to HIV/AIDS. People were asked to pay a certain amount of money if they were caught.*

The margin between those who participated in community activities and those who do not because of the death of a child to HIV/AIDS seems to be quite narrow. The participants made us understand that even though they were not strong enough and did not have enough time they had participated in community activities in order to ensure community support should they need it in the future. They also made us to understand that the community leaders protected their interest. Even though there were very strict roles that people should not avoid those affected by HIV/AIDS or discriminate against them, there were a few cases where people still avoided them because of fear of contamination.

## Discussion

The findings of this study indicate that being an older caregiver of PLWHA not only has a detrimental effect on one's economic/financial well-being; it also negatively influence's one's participation in social/religious and community activities due to the extent of the caregiving responsibilities. The study also documented a fair degree of variation in community reaction reported by participants, and also the roles the community leaders play in protecting the elderly caregivers and the PLWHA.

The data show that older caregivers of PLWHA in Cameroon experience a serious financial burden and a deteriorated quality of life as a direct consequence of the impact of caring for children or grandchildren affected by HIV/AIDS. This finding concurs with similar findings on HIV/AIDS and the elderly in Sub-Saharan Africa (Lekalakala-Mokgele, 2011). Many persons affected by HIV/AIDS remain at home, with the main burden of their care resting almost entirely on family members, who in most cases are elderly females (Ogunmefun & Schatz, 2009). The caregiving role of the elderly is such that it overwhelms their livelihood, forcing them to contend with various demands in terms of coping with increased health care costs, including debts incurred as a result of HIV/AIDS-related illnesses (Kaler, Alibhai, Kipp, Rubaale, & Konde-Lule, 2010; Knodel, 2008), meeting the transport and medical costs of their ailing children (May, 2003), caring for orphaned grandchildren and paying the funeral expenses of their children (Bock & Johnson, 2008; Kipp, Tindyebwa, Rubaale, Karamagi, & Bajenja, 2007).

The study also revealed variations in caregiver participation in social/religious activities. The majority of the participants stated that they had no time or could not attend social/religious activities (such as child naming ceremonies, wedding, traditional marriages, etc.) because they were taking care of a sick child or taking care of their grandchildren. One would have expected to find more elderly people who had children suffering from HIV/AIDS to participate in religious activities to get some solace by seeking support and encouragement from a community of believers, but this apparently is not the case. This finding is in line with other studies which revealed that increased social isolation of the elderly is due to prolonged travelling and absence from their homes to care for sick and orphaned grandchildren (Schatz, 2007; Ssengonzi, 2009). This results in increased social isolation of the elderly because they cannot afford the time or money to take part in social activities (Help Age International, 2008). Another reason for the elderly's reduced participation in social activities is fear of stigmatization, as reported by Christoph et al. (2012).

The findings of this study reveal that most of the participants do participate in community activities (such as cleaning the churches, participating in building community bridges, working in the community leader's farm etc.). Even though they as caregivers are not strong enough and do not have enough time, they still have to participate in these community activities so that in the time of trouble they will get some kind of support from community members. This is contrary to most of the research findings concerning HIV/AIDS and the elderly. In their 2010 study based in rural Kenya, Ice, Yogo, Heh and Juma addressed the impact of older people's participation in community activities. Results in that study provided evidence that parents do not participate in community activities because they spent so much time taking care of their sick child or children orphaned by HIV/AIDS.

The findings also point to a fair degree of variation in community reactions reported by participants. Participants generally indicated neutral or positive responses from community members towards them. Contrary to the common portrayals that emphasize negative responses (Li et al., 2008; Møller & Erstad, 2007), our results reveal that there is a mixture of positive and negative reactions, although the positive support from community members outweighs the negative reactions, though the latter still exist. This finding is supported by that of Knodel et al. (2010) as well as Tanyi and Okoye (2014) who reported that community reaction towards individuals and families suffering from the consequences of AIDS in Thailand, Cambodia and Cameroon appear to be quite positive or neutral for most, but not all.

## Implications

This study has yielded important findings showing that there is a need for government to take more interest in ageing issues, especially when it comes to those concerning HIV/AIDS and its impact on the elderly. These findings have clear implications for caregiving and social policy.

AIDS is still a sensitive matter for many Cameroonian families with an infected member because the issue of social stigma is still very common (Christoph et al., 2012). Trained personnel provide therapy and counselling for concerns such as new diagnoses, disclosure, intimate partner violence, depression, fertility, anxiety, relationships, grief, loss and addictions. These personnel should work within the context of a multidisciplinary team in providing support for those living with a chronic illness. Team members may include a nurse practitioner, physician, pharmacist, psychologist, psychiatrist, immunologist, social workers and a representative from public health.

The number of cumulative AIDS cases in sub-Saharan Africa alone has exceeded 25 million (WHO/UNAIDS, 2013). Human resource personnel's need to be prepared to empower ill and dying patients by maximizing their options to live and to die in the way they choose. To accomplish this crucial clinical task with clients, professionals must feel empowered in their work. The fight for sensitive and effective services for all people with AIDS requires all professionals to help change a small portion of events by not shying away from working with people infected or affected by HIV/AIDS.

The government should target HIV/AIDS programmes for older people. The key to supporting the numerous challenges facing older people as a result of the HIV/AIDS epidemic lies in interventions at community level and in an intergenerational approach. To be effective, such interventions will require collaboration between international agencies and NGOs. The Cameroon government should, for instance, link up with Help Age International who works with community-based organizations such as older people's associations in order to identify and support affected and infected older people at a grassroots level. Help Age International has programmes that combine income generation with support and advice to older caregivers of PLWHA, and to orphans and vulnerable children. Governments and all stakeholders should implement policies and strategies that will provide sufficient physical, emotional and financial support for the elderly to be able to care for the orphaned grandchildren and for themselves.

## Conclusion

The findings of this study indicate that HIV/AIDS places a huge burden on the elderly population's financial well-being, and further impedes on their participation in social/religious activities and community activities due to their caregiving responsibilities. The study also documents varied reactions from the community as reported by participants and community leaders. It is concluded that more needs to be done to articulate the knowledge base of the impact of HIV/AIDS in order to inform social, economic and political policies for the purpose of alleviating the problems that the pandemic is wreaking on the elderly population in Cameroon. Evidence from our study is nevertheless limited to the Mezam, Menchum and Donga/Mantung divisions in the Northwest region of Cameroon. Caution should, therefore, be exercised when drawing conclusions from our findings and applying them to other regions not included in this study.

## Appendix 1. FGDs guide for grandparent

**A. Introduction:** Welcome participants

Describe what FGD is – a group discussion that allows you to discuss the topic among yourselves rather than talking to us.

**B. Purpose/Modus Operandi:**

We will be discussing HIV/AIDS Disease burden on the elderly population.

We are interested in all your ideas, comments and suggestions.

All comments both positive and negative are welcome.

Please feel free to disagree with one another. We would like to have many points of view.

(We would want you to discuss all the issues among yourselves).

(Explain the use of audio tape) All comments are confidential and for research purposes only.

We would also want you to speak one at a time so that the tape recorder can pick your voice appropriately and clearly.

**Sources of Support**

What are your major sources of support?

**Probe**:

Whether relative, children support or others are the people that support most

Whether he has personal support system like pension and savings. Relationship with grand children

1. How many children do you have? Probe whether they are alive or dead of HIV/AIDS.
2. How many of your children are married? Probe whether they had children before they died or fell sick
3. How many grandchildren do you have? Probe. To find out where the children are staying and why?
4. Do you give your grandchildren any support? Probe for the type of support they give the grandchildren.
5. Before your adult child became sick or dead, does he/she contribute to family income?
6. How has the fact that your sick or dead child not contributing to family income made your financial situation worst
7. By your own estimation, how would you rate your financial status? Probe for before the dead or sickness of the child.

**Psychological wellbeing of older adult parents**

1. Parents were asked if they have any ill feeling after the death of their child to HIV/AIDS

**Probe**

1. Did you have any psychological pain of seeing your child suffering and dying of HIV/AIDS?
2. Do you have any feeling by caregiving demands? Maybe form you sick child or from your grandchildren?
3. Did you have any psychological pain from anticipated or enacted negative community reaction?
4. Do you have any anxiety concerning consequences for financial security?

**Membership and Participation in religious/ social activities**

Frequency of participation in various social activities

Types of social group that the participants belong to

Types of social activities that the participants engage in

Frequency of attendance

Reasons for not attending regularly

1. Do you participate in any religious /social activities? Probe to fine out the type of social activity she attends. Also probe to know if she does not attend and why she does not attend.
2. If you participate in any social activities, how often do you attend their meetings?
3. Are you a member of any religious organization? Probe to know the organization and whether she attends meetings. And if not why does she not attend meetings.

**Participation in community activities**

1. Do you participate in community activity? Probe to know whether they participate, or not. Also probe to know how often they participate and also probe if they participate to know why?

Funeral are very important social events in Cameroon that reflects the prestige and reputation of the family within the community. They typically last at least several days and involve treating guest with refreshments and meals. The expenses are incurred all at once unlike cost of care and treatment that may be spread out over the period of illness.

**Probe**

1. Did you have to spend a lot of money for h/she funeral?
2. When he died, did any one help you at his funeral at all?
3. Did you have to sale any property to meet up with some of the funeral expenses?
4. Did you think that the funeral expenses were a burden to you and your family?
5. Did you have to borrow money from someone to cover the expenses due to his illness and death?
6. Did you have any difficulty taking care of the children he/she left behind?

**Thanks.**

## Appendix 2. IDI guide for community leaders

*The HIV/AIDS Disease burden on the elderly population in Cameroon*

Preliminaries

Introduce yourself and the purpose of the interview

Purpose/Modus Operandi:

We will be discussing HIV/AIDS.

We are interested in all your ideas, comments and suggestions.

All comments both positive and negative are welcome.

(Explain the use of the audio tape) All comments are confidential and for research purposes only).

**1. Treatment of HIV/AIDS**

Do you think HIV/AIDS is a threat to this community?
How big a threat is HIV/AIDS to the community?
What group does HIV/AIDS affect most in this community?

**Probe**:

By age groups and sex (children, male youths, female youths, women in the reproductive age group, adult males and the elderly)

How do people in this community treat people with HIV/AIDS?

As a community leader what punishment do you give to people who abuse the HIV/AIDS affected people in your community?

**2. Prevention of HIV/AIDS**

What are some of the things that members of this community do to prevent HIV/AIDS?

**3. What is the impact of HIV/AIDS?**

On the individual

On the family

On the community/neighborhood

**4. Community efforts towards care given to the people affected by HIV/AIDS**

When someone dies of HIV/AIDS, who takes care of the children, the parents and other siblings?

Are there any community efforts to care for children orphaned by HIV/AIDS? **Thank you**
